# Factors Associated With Childhood Asthma and Wheeze in Chinese Preschool-Aged Children

**DOI:** 10.3389/fmed.2021.742581

**Published:** 2021-11-04

**Authors:** Xiangling Deng, Min Yang, Shunan Wang, Qiong Wang, Bo Pang, Kundi Wang, Zhixin Zhang, Wenquan Niu

**Affiliations:** ^1^Graduate School, Beijing University of Chinese Medicine, Beijing, China; ^2^International Medical Services, China-Japan Friendship Hospital, Beijing, China; ^3^Department of Pediatrics, China-Japan Friendship Hospital, Beijing, China; ^4^Institute of Clinical Medical Sciences, China-Japan Friendship Hospital, Beijing, China

**Keywords:** childhood asthma, wheeze, preschool-aged children, risk factors, LASSO regression, nomogram

## Abstract

This study was prepared to identify and characterize potential factors associated with childhood asthma and wheeze in Chinese preschool-aged children. A comprehensive questionnaire was designed for children aged 3–6 years and their parents or guardians in Beijing and Tangshan from September to December 2020. The least absolute shrinkage and selection operator (LASSO) model was used to identify factors in a significant association with childhood asthma and wheeze, respectively. The LASSO model was internally validated using bootstrap resampling with 100 replications. A total of 9,529 questionnaires were certified as eligible for inclusion after stringent quality control. The prevalence of doctor-diagnosed childhood asthma and parent-reported wheeze was 2.8 and 6.2%, respectively. Factors simultaneously associated with childhood asthma and wheeze were children with a history of allergic rhinitis, hay fever, eczema, initial age of using antibiotics, body mass index category, and family history of asthma. Specifically, children's vitamin D supplement duration was significantly associated with childhood asthma, whereas the association with childhood wheeze was significant for intake frequency of night meals for children and their screen time. Modeling of significant factors in nomograms had decent prediction accuracies, with C-index reaching 0.728 and 0.707 for asthma and wheeze, respectively. In addition, internal validation was good, with bootstrap C-statistic of being 0.736 for asthma and 0.708 for wheeze. Taken together, our findings indicated that the development of asthma and wheeze among preschool-aged children was probably determined by the joint contribution of multiple factors including inherited, nutritional, unhealthy lifestyles, and history of allergic disease. Further validation in other groups is necessary.

## Introduction

Asthma is a heterogeneous disease characterized by chronic airway inflammation, and it is defined by the history of respiratory symptoms, such as wheeze, chest tightness, shortness of breath, and cough that are varying over time and in intensity, together with variable expiratory airflow limitation (1). Asthma has become the most common non-communicable disease among children (2), and it imposes a heavy burden on health and healthcare systems (3). According to the reports of the International Study of Asthma and Allergies in Childhood (ISAAC), the prevalence of asthma symptoms has exhibited an increasing tendency globally in both children (from 11.1 to 11.6%) and adolescents (13.2–13.7%). Preschool-aged children have a relatively high asthma-related morbidity, with about 48% of preschool-aged children with asthma experiencing an asthma attack in the preceding year (4). Meanwhile, a wide variation existed in the symptom prevalence of childhood asthma worldwide (5). Compared with some developed countries, such as Austria (11.3%), the United States (8.7%), the United Kingdom (15.4%), and Canada (13%) (6, 7), the prevalence of childhood asthma in China was much lower. The 3rd Nationwide Survey of Childhood Asthma in Urban Areas of China recorded that the prevalence of asthma in children under 14 years had been increased to 3% in 2020 from 2% in 2000.

It is worth noting that childhood asthma is preventable. Asthma usually starts before school age, and approximately one-third to half of the children with moderate to severe asthma may persist into adulthood (8). As evidenced, childhood asthma may predispose people to chronic obstructive pulmonary disease (9). Much worse, a proportion of children with asthma had severe symptoms and frequent exacerbations even with the availability of effective drugs (10). It is hence necessary to gain a better understanding of factors responsible for the development of childhood asthma and to inform alternative public health strategies that can effectively reduce or control the prevalence of asthma worldwide.

To yield more information, we designed a large-scale cross-sectional survey in Beijing and Tangshan, aiming to identify and characterize potential factors associated with childhood asthma and wheeze in Chinese preschool-aged children.

## Methods

### Study Design

During the period between September and December in 2020, we undertook a cross-sectional survey of preschool children from Beijing and Tangshan, according to the principles of the Declaration of Helsinki. The survey was reviewed and approved by the Ethics Committee of China–Japan Friendship Hospital and all parents or guardians of study children read and signed informed consent forms prior to participation.

### Study Children

In this survey, study subjects were restricted to preschool-aged children (3–6 years old) who attended junior to senior classes in kindergartens. We utilized a stratified cluster random sampling strategy in 16 districts from Beijing and one city (Tangshan) in Hebei. In total, 30 kindergartens were eligible for data collection.

### Basic Characteristics and Quality Control

A comprehensive questionnaire was self-designed for both children and their parents or guardians, aiming to collect potential factors responsible for the development of childhood asthma and wheeze. Items from the questionnaire were selected *a priori* based upon published literature and clinical experience.

From children, items of interest included age, sex, region, body mass index (BMI), birth weight, ABO blood type, delivery mode, breastfeeding duration, daily sleep duration, secondhand smoke exposure, vitamin D supplement duration, probiotics supplement, exposure of average daily screen time, intake frequency of night meals, history of allergic rhinitis, hay fever, eczema, and initial age of using antibiotics. Body weight (to the nearest 0.1 kg) and height (to the nearest 0.1 cm) were measured by trained health physicians.

From parents or guardians, data on age, sex, weight, height, education, family income, maternal pre-pregnancy weight, gestational weight gain (GWG), maternal pregnancy smoking exposure, gestational hypertension, gestational diabetes mellitus, and family history of asthma were self-reported and recorded simultaneously.

Kindergarten teachers from 30 designated kindergartens were responsible for explaining and circulating the questionnaires to the parents or guardians of all children. Children with a history of the disease, including chronic kidney disease, hypothyroidism, congenital heart disease, chronic respiratory diseases, and inherited metabolic diseases, were also eliminated. Information on chronic medical histories of study children was obtained from their latest health records.

### Definition of Characteristics

For children, asthma was defined according to the records of doctors, and wheezing symptoms were collected based on parental reports, including the onset of wheeze during the past 12 months. BMI was calculated and divided into obesity, overweight, and non-overweight according to the China criteria (2009) (11). Both break time during the day and sleep time at night was summed to calculate sleep duration of children, that is, sleep time on working days × 5 and corresponding time on weekends × 2 divided by 7. Average daily screen time was calculated as the same as the daily sleep duration and grouped into four groups, *viz*. <0.5, 0.5–1, 1–1.5, and >1.5 h. Intake frequency of night meals was defined as weekly eating food within 2 h before bedtime, which was classified as often (≥3 times), occasional (one to two times), once in a while, and none. Vitamin D supplement duration was classified into four categories, *viz*. ≤3, 3–6, 6–12, and >12 months. The initial age of using antibiotics was divided into seven groups, that is, <28 days, 28 days−1 month, 1–6 months, 6 months−1 year, 1–3 years, 3–6 years, and without antibiotics in the first 6 years of life.

For parents, family income (RMB per year) was classified into ≤100,000, 100,000–300,000, 300,000–600,000, 600,000–900,000, and >1,000,000. Education was defined as a high school degree or below, college degree, master's degree, and doctor degree or above. Adequate GWG was defined as weight gain of 12.5–18 kg in underweight mothers, 11.5–16 kg in normal-weight mothers, 7–11.5 kg in overweight mothers, and 5–9 kg in obese mothers. Inadequate GWG was grouped as less than the lower limits of adequate levels, and excessive GWG meant greater than the upper limits of adequate levels. All reference criteria were based on the recommendations of the Institute of Medicine (2009) (12). Secondhand smoke exposure and maternal smoking were defined as smoking exposure during maternal pregnancy.

### Statistical Analyses

Statistical analyses were completed using the STATA software (version 14.0, Stata Corp., College Station, TX, USA) and the R programming environment (version 4.0.2, https://www.r-project.org/). The power to detect statistical significance was estimated by the PS-Power Simple Size software (version 3.1.2, Copyright 1997–2009 by William D. Dupont and Walton D. Plummer.).

As data under study were collected from preschool children from 30 kindergartens, intraclass correlation coefficient (ICC) was calculated to quantify the degree to which observations within a cluster differ from those between clusters (13). The ICC statistic ranges from 0 to 1, with a lower value indicating a lower likelihood of between-kindergarten variation.

Continuous data are expressed as median (interquartile range), and categorical data are summarized as count (percentage). Two-group comparison was done by using the Wilcoxon rank-sum test or χ^2^ test, when appropriate. Two-sided *p* < 5% was defined to be statistically significant.

To account for missing data, we used multiple imputations, based on five replications and a chained equation approach method in the R MI procedure (14). All children were divided into asthmatic/wheezing or non-asthmatic groups.

To minimize the effects of potential confounding factors including age, sex, region, blood type, delivery mode, and family income, a 1:3 propensity score matching (PSM) method was performed to compare the relationship of various factors and these two groups by using the psmatch2 routine in the STATA software (Stata Corp., College Station, TX, USA).

The least absolute shrinkage and selection operator (LASSO) model (15) was used to quantify the contribution of all possible factors in this survey. LASSO is effective in declining covariance between multiple factors, reducing the possibility of over-fitting and removing unnecessary covariates. The “glmnet” package (version 2.0-16, https://glmnet.stanford.edu) was used to fit the logistic LASSO regression, and 10-fold cross-validation was used to select the penalty term, λ. Selected factors of statistical significance formed the elements of the prediction model.

The R “rms” package was used to create a prediction nomogram model based on selected factors of significance for the early identification of childhood asthma or wheeze. The area under the receiver operating characteristic (ROC) curve (AUC) was calculated to evaluate the discrimination ability of nomogram models. The predictive accuracy of nomogram models was determined using both calibration plots and C-index.

### Internal Validation of Nomogram Models

Models for predicting childhood asthma and wheeze were internally validated using bootstrap resampling with 100 replications (16, 17). For each step of resampling, models were refitted, and model discrimination and calibration were assessed on bootstrapped data and validated on original data. The difference in performance between the two datasets was calculated and averaged over the 100 replications to calculate optimism-adjusted C-statistics.

## Results

### Baseline Characteristics

In total, our questionnaires were sent to the parents or guardians of 10,441 children, and the response rate was 98% (*n* = 10,230) within the scheduled time. Finally, 9,529 questionnaires were certified as eligible for inclusion after strict quality control. In addition, the ICC statistics for all study factors were lower than 0.05, indicating a lower likelihood of clustering across kindergartens.

The baseline characteristics of 9,529 children in the present study are shown in Table 1. The prevalence of childhood asthma and wheeze was 2.8% and 6.2%, respectively. After PSM, for asthma, the number of children with asthma and without was 265 and 752, separately. For wheeze, the number of children with asthma and without was 594 and 1,611, severally.

**Table 1:** Baseline characteristics of study participants in this study.

**Characteristics**	**Asthma**		***P***	**Wheeze**		***P***
	**No** **(***n*** = 9,264)**	**Yes** **(***n*** = 265)**		**No** **(***n*** = 8,773)**	**Yes** **(***n*** = 747)**	
**For children**						
Age, years	4.6 (4.0, 5.6)	4.8 (4.2, 5.8)	<0.001	4.6 (4.0, 5.6)	4.6 (4.0, 5.6)	0.212
Gender			<0.001			0.002
Boys	4,716 (50.9%)	168 (63.4%)		4,459 (50.8%)	425 (56.9%)	
Girls	4,548 (49.1%)	97 (36.6%)		4,314 (49.2%)	322 (43.1%)	
Region			<0.001			<0.001
Beijing	6,042 (65.2%)	202 (76.5%)		5,667 (64.6%)	568 (76%)	
Hebei	3,223 (34.8%)	62 (23.5%)		3,106 (35.4%)	179 (24%)	
ABO blood types			<0.001			0.979
A	1,563 (27.8%)	52 (29.2%)		1,471 (27.9%)	144 (28.1%)	
B	1,912 (34.1%)	76 (42.7%)		1,810 (34.3%)	178 (34.7%)	
O	1,668 (29.7%)	40 (22.5%)		1,557 (29.5%)	151 (29.4%)	
AB	470 (8.4%)	10 (5.6%)		440 (8.3%)	40 (7.8%)	
Premature delivery			0.158			0.279
Yes	806 (8.9%)	30 (11.4%)		762 (8.8%)	74 (10%)	
No	8298 (91.1%)	234 (88.6%)		7,867 (91.2%)	665 (90%)	
Birth weight (kg)			0.400			0.521
Normal	7,998 (87%)	221 (84.4%)		7,579 (87%)	640 (86.3%)	
Small for gestational age	361 (3.9%)	11 (4.2%)		337 (3.9%)	35 (4.7%)	
Large for gestational age	834 (9.1%)	30 (11.5%)		797 (9.1%)	67 (9%)	
Delivery mode			0.11			0.383
Natural delivery	4,935 (53.3%)	128 (48.3%)		4,677 (53.3%)	386 (51.6%)	
Cesarean section	4,329 (46.7%)	137 (51.7%)		4,104 (46.7%)	362 (48.4%)	
BMI (kg/m^2^)	15.4 (14.5,16.6)	15.7 (14.8, 17.4)	0.011	15.4 (14.5, 16.6)	15.7 (14.6, 17.0)	<0.001
BMI category			0.062			0.011
Non overweight or obesity	3,194 (68.3%)	99 (60.4%)		3,028 (68.5%)	265 (63.2%)	
Overweight	768 (16.4%)	30 (18.3%)		730 (16.5%)	68 (16.2%)	
Obesity	715 (15.3%)	35 (21.3%)		664 (15%)	86 (20.5%)	
Breastfeeding duration (months)	12.0 (8.0, 18.0)	12.0 (6.0, 18.0)	0.023	12.0 (8.0, 18.0)	12.0 (8.0, 18.0)	0.217
Sleep duration	10.0 (9.0, 10.6)	9.7 (9.0, 10.3)	0.004	10.0 (9.0, 10.6)	10.0 (9.0, 10.6)	0.919
Intake frequency of night meals			0.507			<0.001
None or once in a while	5,454 (58.9%)	146 (55.1%)		5246 (59.7%)	354 (47.3%)	
1–2 times weekly	2,127 (23.0%)	66 (24.9%)		1,984 (22.6%)	209 (27.9%)	
3–5 times weekly	884 (9.5%)	31 (11.7%)		819 (9.3%)	96 (12.8%)	
Every day	799 (8.6%)	22 (8.3%)		732 (8.3%)	89 (11.9%)	
Secondhand smoke exposure			0.426			<0.001
No	5,276 (57%)	149 (56.2%)		5,020 (57.2%)	405 (54.1%)	
1–5 cigarettes per day	2,621 (28.3%)	68 (25.7%)		2,499 (28.5%)	190 (25.4%)	
5–10 cigarettes per day	817 (8.8%)	30 (11.3%)		767 (8.7%)	80 (10.7%)	
>10 cigarettes per day	550 (5.9%)	18 (6.8%)		495 (5.6%)	73 (9.8%)	
Vitamin D supplement duration			0.920			0.011
≤3 months	1,873 (23.3%)	58 (24.0%)		1,804 (23.8%)	127 (18.5%)	
3–6 months	1,374 (17.1%)	43 (17.8%)		1,306 (17.2%)	111 (16.2%)	
6–12 months	1,637 (20.4%)	49 (20.2%)		1,538 (20.3%)	148 (21.5%)	
>12 months	3,155 (39.2%)	92 (38%)		2,946 (38.7%)	301 (43.8%)	
Probiotics supplemented			0.002			<0.001
Yes	6,318 (68.2%)	205 (77.4%)		5,917 (67.4%)	606 (81%)	
No	2,946 (31.8%)	60 (22.6%)		2,864 (32.6%)	142 (19%)	
Screen time (h/per day)			0.962			0.034
<0.5 h	1,813 (19.6%)	49 (18.6%)		1,727 (19.7%)	135 (18.1%)	
0.5–1 h	3,414 (37.0%)	101 (38.4%)		3,266 (37.3%)	249 (33.4%)	
1–1.5 h	1,580 (17.1%)	45 (17.1%)		1,485 (17%)	140 (18.8%)	
>1.5 h	2,424 (26.3%)	68 (25.9%)		2,271 (26%)	221 (29.7%)	
Allergic rhinitis			<0.001			<0.001
Yes	1,460 (15.8%)	125 (47.2%)		1,284 (14.6%)	301 (40.2%)	
No	7,804 (84.2%)	140 (52.8%)		7,497 (85.4%)	447 (59.8%)	
History of hayfever			<0.001			<0.001
Yes	297 (3.2%)	41 (15.5%)		242 (2.8%)	96 (12.8%)	
No	8,967 (96.8%)	224 (84.5%)		8,539 (97.2%)	652 (87.2%)	
History of eczema			<0.001			<0.001
Yes	2,946 (31.8%)	125 (47.2%)		2,658 (30.3%)	413 (55.2%)	
No	6,318 (68.2%)	140 (52.8%)		6,123 (69.7%)	335 (44.8%)	
Initial age of antibiotic use			<0.001			<0.001
<28 days	592 (6.4%)	11 (4.2%)		562 (6.4%)	41 (5.5%)	
28 days−1 month	101 (1.1%)	7 (2.6%)		93 (1.1%)	15 (2%)	
1 month−6 months	741 (8.0%)	34 (12.8%)		683 (7.8%)	92 (12.3%)	
6 months−1 year	1,618 (17.5%)	54 (20.4%)		1,492 (17%)	180 (24.1%)	
1 year−3 years	3,815 (41.2%)	118 (44.5%)		3,615 (41.2%)	318 (42.5%)	
3 years−6 years	1,334 (14.4%)	28 (10.6%)		1,284 (14.6%)	78 (10.4%)	
Never used	1,063 (11.5%)	13 (4.9%)		1,052 (12%)	24 (3.2%)	
**For parents or guardians**						
Family income (RMB per year)			0.002			<0.001
≤100,000	3,176 (34.3%)	66 (24.9%)		3,060 (34.8%)	182 (24.3%)	
100,000–300,000	3,443 (37.2%)	97 (36.6%)		3,237 (36.9%)	303 (40.5%)	
300,000–600,000	1,842 (19.9%)	68 (25.7%)		1,724 (19.6%)	186 (24.9%)	
600,000–900,000	514 (5.5%)	20 (7.5%)		484 (5.5%)	50 (6.7%)	
>1000,000	289 (3.1%)	14 (5.3%)		276 (3.1%)	27 (3.6%)	
Parental education			0.024			<0.001
High school degree or below	3,399 (36.7%)	73 (27.5%)		3,273 (37.3%)	199 (26.6%)	
College degree	4,369 (47.2%)	145 (54.7%)		4,110 (46.8%)	404 (54%)	
Master degree	1,065 (11.5%)	33 (12.5%)		995 (11.3%)	103 (13.8%)	
Doctor degree and above	431 (4.7%)	14 (5.3%)		403 (4.6%)	42 (5.6%)	
Elderly parturient women			0.282			0.193
Yes	1223 (13.3%)	29 (11%)		1165 (13.4%)	87 (11.7%)	
No	7,966 (86.7%)	234 (89%)		7,543 (86.6%)	657 (88.3%)	
Gestational hypertension			0.091			<0.001
Yes	747 (8.1%)	29 (10.9%)		688 (7.8%)	88 (11.8%)	
No	8,517 (91.9%)	236 (89.1%)		8,093 (92.2%)	660 (88.2%)	
Gestational diabetes mellitus			0.565			0.536
Yes	228 (2.5%)	8 (3%)		220 (2.5%)	16 (2.1%)	
No	9,036 (97.5%)	257 (97%)		8,561 (97.5%)	732 (97.9%)	
Family history of asthma			<0.001			<0.001
Yes	434 (4.7%)	40 (15.1%)		395 (4.5%)	79 (10.6%)	
No	8,830 (95.3%)	225 (84.9%)		8,386 (95.5%)	669 (89.4%)	
Pre-pregnant obesity			0.738			0.378
Yes	424 (4.6%)	11 (4.2%)		396 (4.5%)	39 (5.2%)	
No	8,822 (95.4%)	254 (95.8%)		8,368 (95.5%)	708 (94.8%)	
Gestational weight gain (kg/m^2^)			0.503			0.139
Inadequate	2,355 (25.4%)	59 (22.3%)		2,243 (25.5%)	171 (22.9%)	
Adequate	3,150 (34%)	93 (35.1%)		2,993 (34.1%)	250 (33.4%)	
Excessive	3,759 (40.6%)	113 (42.6%)		3,545 (40.4%)	327 (43.7%)	
Smoking exposure during maternal pregnancy			0.177			<0.001
Yes	5,883 (63.5%)	179 (67.5%)		5,537 (63.1%)	525 (70.2%)	
No	3,381 (36.5%)	86 (32.5%)		3,244 (36.9%)	223 (29.8%)	

*BMI, body mass index. Data are expressed as median (interquartile range) or count (percent). P-value was calculated by the rank-sum test or the χ^2^ test, where appropriate*.

### Identification of Potential Factors

As shown in Table 2, significant differences between children with and without asthma were observed for probiotics supplement, allergic rhinitis, history of hay fever, eczema, initial age of using antibiotics, and family history of asthma (*p* ≤ 0.05). Univariate analyses suggested statistically significant associations of sex, BMI category, intake frequency of night meals, probiotics supplement, screen time, allergic rhinitis, history of hay fever, eczema, initial age of using antibiotics, and family history of asthma with childhood wheeze.

**Table 2:** Baseline characteristics of study participants after propensity score matching in this study.

**Characteristics**	**Asthma**		***P***	**Wheeze**		***P***
	**Without (*n* = 752)**	**With (*n* = 265)**		**Without (*n* = 1,611)**	**With (*n* = 594)**	
**For children**						
Age, years	5 (4.2, 5.9)	4.8 (4.2, 5.8)	0.319	4.6 (4.0, 5.7)	4.6 (4.0, 5.6)	0.387
Gender						
Boys	471 (62.6%)	168 (63.4%)	0.825	783 (48.6%)	330 (55.6%)	0.004
Girls	281 (37.4%)	97 (36.6%)		828 (51.4%)	264 (44.4%)	
Region			0.326			0.266
Beijing	553 (73.5%)	203 (76.6%)		1,166 (72.4%)	444 (74.7%)	
Hebei	199 (26.5%)	62 (23.4%)		445 (27.6%)	150 (25.3%)	
ABO blood types			0.721			0.982
A	266 (35.4%)	86 (32.5%)		469 (29.1%)	170 (28.6%)	
B	252 (33.5%)	97 (36.6%)		566 (35.1%)	206 (34.7%)	
O	200 (26.6%)	68 (25.7%)		438 (27.2%)	166 (27.9%)	
AB	34 (4.5%)	14 (5.3%)		138 (8.6%)	52 (8.8%)	
Premature delivery			0.280			0.067
Yes	68 (9%)	30 (11.3%)		126 (7.8%)	61 (10.3%)	
No	684 (91%)	235 (88.7%)		1485 (92.2%)	533 (89.7%)	
Birth weight (kg)			0.422			0.843
Normal	659 (87.6%)	224 (84.5%)		1,391 (86.3%)	509 (85.7%)	
Small for gestational age	27 (3.6%)	11 (4.2%)		72 (4.5%)	30 (5.1%)	
Large for gestational age	66 (8.8%)	30 (11.3%)		148 (9.2%)	55 (9.3%)	
Delivery mode			0.462			0.741
Natural delivery	369 (49.1%)	137 (51.7%)		847 (52.6%)	317 (53.4%)	
Cesarean section	383 (50.9%)	128 (48.3%)		764 (47.4%)	277 (46.6%)	
BMI category			0.310			0.016
Non overweight or obesity	504 (67%)	164 (61.9%)		1,092 (67.8%)	370 (62.3%)	
Overweight	121 (16.1%)	48 (18.1%)		263 (16.3%)	100 (16.8%)	
Obesity	127 (16.9%)	53 (20%)		256 (15.9%)	124 (20.9%)	
Breastfeeding duration (months)	9.7 (9, 10.3)	12 (8,18)	0.051	12 (8,18)	12.5 (8,18)	0.453
Sleep duration	10 (9, 10.4)	9.7 (9, 10.3)	0.090	10 (9, 10.6)	10 (9.3, 10.6)	0.148
Intake frequency of night meals			0.612			<0.001
None or once in a while	437 (58.1%)	146 (55.1%)		917 (56.9%)	277 (46.6%)	
1–2 times weekly	183 (24.3%)	66 (24.9%)		407 (25.3%)	163 (27.4%)	
≥3 times weekly	132 (17.6%)	53 (20%)		287 (17.8%)	154 (25.9%)	
Secondhand smoke exposure			0.886			0.153
Yes	333 (44.3%)	116 (43.8%)		683 (42.4%)	272 (45.8%)	
No	419 (55.7%)	149 (56.2%)		928 (57.6%)	322 (54.2%)	
Vitamin D supplement duration			0.418			0.071
≤3 months	54 (7.2%)	16 (6%)		93 (5.8%)	24 (4%)	
3–6 months	105 (14%)	50 (18.9%)		252 (15.6%)	78 (13.1%)	
6–12 months	142 (18.9%)	49 (18.5%)		290 (18%)	94 (15.8%)	
>12 months	155 (20.6%)	50 (18.9%)		346 (21.5%)	135 (22.7%)	
Probiotics supplemented			<0.001			<0.001
Yes	495 (65.8%)	205 (77.4%)		1,103 (68.5%)	483 (81.3%)	
No	257 (34.2%)	60 (22.6%)		508 (31.5%)	111 (18.7%)	
Screen time (h/per day)			0.829			<0.001
<0.5 h	153 (20.3%)	49 (18.5%)		340 (21.1%)	105 (17.7%)	
0.5–1 h	270 (35.9%)	102 (38.5%)		638 (39.6%)	194 (32.7%)	
1–1.5 h	137 (18.2%)	45 (17%)		263 (16.3%)	114 (19.2%)	
>1.5 h	192 (25.5%)	69 (26%)		370 (23%)	181 (30.5%)	
Allergic rhinitis			<0.001			<0.001
Yes	128 (17%)	125 (47.2%)		264 (16.4%)	218 (36.7%)	
No	624 (83%)	140 (52.8%)		1,347 (83.6%)	376 (63.3%)	
History of hayfever			<0.001			<0.001
Yes	30 (4%)	41 (15.5%)		55 (3.4%)	68 (11.4%)	
No	722 (96%)	224 (84.5%)		1,556 (96.6%)	526 (88.6%)	
History of eczema			<0.001			<0.001
Yes	257 (34.2%)	125 (47.2%)		493 (30.6%)	328 (55.2%)	
No	495 (65.8%)	140 (52.8%)		1,118 (69.4%)	266 (44.8%)	
Initial age of using antibiotics			<0.001			<0.001
<28 days	56 (7.4%)	11 (4.2%)		187 (11.6%)	18 (3%)	
28 days-1 month	9 (1.2%)	7 (2.6%)		233 (14.5%)	64 (10.8%)	
1 month−6 months	60 (8%)	34 (12.8%)		657 (40.8%)	252 (42.4%)	
6 months−1 year	110 (14.6%)	54 (20.4%)		303 (18.8%)	144 (24.2%)	
1 year−3 years	306 (40.7%)	118 (44.5%)		119 (7.4%)	66 (11.1%)	
3 years−6 years	119 (15.8%)	28 (10.6%)		19 (1.2%)	11 (1.9%)	
Never used	92 (12.2%)	13 (4.9%)		93 (5.8%)	39 (6.6%)	
**For parents or guardians**						
Family income (RMB per year)			0.945			0.384
≤100,000	203 (27%)	66 (24.9%)		462 (28.7%)	150 (25.3%)	
100,000–300,000	274 (36.4%)	97 (36.6%)		652 (40.5%)	242 (40.7%)	
300,000–600,000	179 (23.8%)	68 (25.7%)		358 (22.2%)	147 (24.7%)	
600,000–900,000	60 (8%)	20 (7.5%)		86 (5.3%)	38 (6.4%)	
>1000,000	36 (4.8%)	14 (5.3%)		53 (3.3%)	17 (2.9%)	
Parental education			0.547			0.263
High school degree or below	243 (32.3%)	73 (27.5%)		504 (31.3%)	161 (27.1%)	
College degree	385 (51.2%)	145 (54.7%)		834 (51.8%)	320 (53.9%)	
Master degree	85 (11.3%)	33 (12.5%)		198 (12.3%)	81 (13.6%)	
Doctor degree and above	39 (5.2%)	14 (5.3%)		75 (4.7%)	32 (5.4%)	
Elderly parturient women			0.177			0.600
Yes	107 (14.2%)	29 (10.9%)		206 (12.8%)	71 (12%)	
No	645 (85.8%)	236 (89.1%)		1,405 (87.2%)	523 (88%)	
Gestational hypertension			0.654			0.090
Yes	75 (10%)	29 (10.9%)		153 (9.5%)	71 (12%)	
No	677 (90%)	236 (89.1%)		1,458 (90.5%)	523 (88%)	
Gestational diabetes mellitus			0.493			0.931
Yes	17 (2.3%)	8 (3%)		39 (2.4%)	14 (2.4%)	
No	735 (97.7%)	257 (97%)		1,572 (97.6%)	580 (97.6%)	
Family history of asthma			<0.001			<0.001
Yes	21 (2.8%)	40 (15.1%)		75 (4.7%)	52 (8.8%)	
No	731 (97.2%)	225 (84.9%)		1536 (95.3%)	542 (91.2%)	
Pre-pregnant obesity			0.942			0.155
Yes	32 (4.3%)	11 (4.2%)		69 (4.3%)	34 (5.7%)	
No	720 (95.7%)	254 (95.8%)		1,542 (95.7%)	560 (94.3%)	
Gestational weight gain (kg/m^2^)			0.135			0.336
Inadequate	215 (28.6%)	59 (22.3%)		387 (24%)	140 (23.6%)	
Adequate	246 (32.7%)	93 (35.1%)		580 (36%)	197 (33.2%)	
Excessive	291 (38.7%)	113 (42.6%)		644 (40%)	257 (43.3%)	
Smoking exposure during maternal pregnancy			0.531			0.074
Yes	492 (65.4%)	179 (67.5%)		1,083 (67.2%)	423 (71.2%)	
No	260 (34.6%)	86 (32.5%)		528 (32.8%)	171 (28.8%)	

*BMI, body mass index. Data are expressed as median (interquartile range) or count (percent). P-value was calculated by the rank-sum test or the χ^2^ test, where appropriate*.

**Figure 1:**
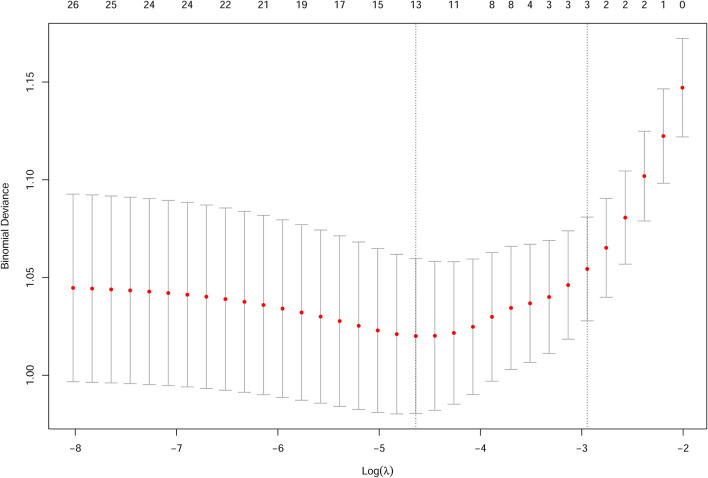
Cross validation plot for the penalty term for childhood asthma.

Table 3 shows the estimated coefficients for candidate factors with childhood asthma and wheeze in the logistic LASSO regression analysis. For doctor-diagnosed childhood asthma, the λ-values ranged from 0.000328 to 0.1344, and the opted λ-value was 0.02475 in this study (Figure 1). Finally, the LASSO regression analysis revealed that seven factors contributed to the development of childhood asthma, including children with a history of allergic rhinitis (β = 1.35), hay fever (β = 0.88), eczema (β = 0.34), initial age of using antibiotics (β = 0.10), vitamin D supplement duration (β = −0.19), BMI category (β = 0.24), and family history of asthma (β = 1.81).

**Table 3:** The estimated coefficients for logistic least absolute shrinkage and selection operator (LASSO) regression between candidate risk factors with doctor-diagnostic childhood asthma and parent-reported wheeze.

**Asthma**		**Wheeze**	
**Variables**	**Coefficients (SE)**	**Variables**	**Coefficients (SE)**
Age	−0.25 (0.09)	Age	−0.10 (0.06)
Allergic rhinitis	1.35 (0.18)	Allergic rhinitis	0.79 (0.12)
History of hay fever	0.88 (0.29)	History of hay fever	0.70 (0.21)
History of eczema	0.34 (0.16)	History of eczema	0.84 (0.10)
Age of first antibiotic using	0.10 (0.05)	Age of first antibiotic using	0.15 (0.04)
Vitamin D supplement duration	−0.19 (0.06)	Night meals intake frequency	0.21 (0.06)
BMI category	0.24 (0.10)	BMI category	0.20 (0.06)
Family history of asthma	1.81 (0.30)	Family history of asthma	0.58 (0.20)
		Screen time (h/per day)	0.21 (0.05)

**Figure 2:**
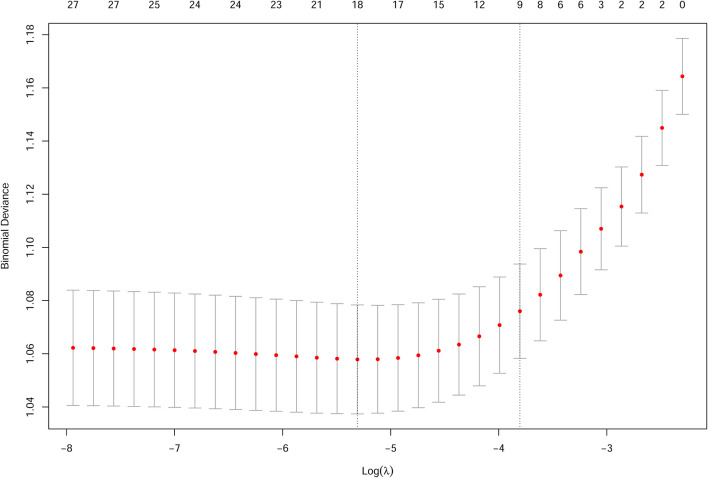
Cross validation plot for the penalty term for childhood wheeze.

For parent-reported wheeze, the λ-values ranged from 0.1002 to 0.000356, and the opted λ-value was 0.02689 (Figure 2). Eight factors were selected to be significantly associated with childhood wheeze, including children with a history of allergic rhinitis (β = 0.79), hay fever (β = 0.70), eczema (β = 0.84), initial age of using antibiotics (β = 0.15), intake frequency of night meals (β = 0.21), BMI category (β = 0.20), family history of asthma (β = 0.58), and screen time (h/day) (β = 0.21). The plots for LASSO regression coefficients over different values of the penalty parameter could be seen in Supplementary Figure 1.

For each selected factor associated with childhood asthma and wheeze, the power to detect significance was consistently over 90%, indicating the robustness of our findings.

### Risk Prediction Nomogram Models

**Figure 3:**
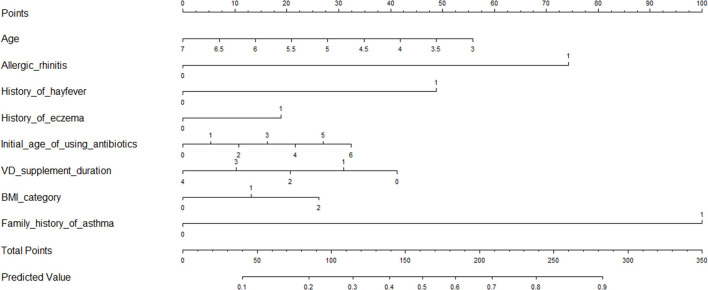
Prediction nomogram for prediction in children with asthma.

**Figure 4:**
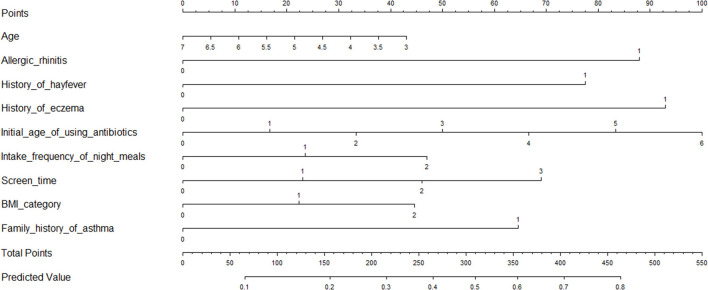
Prediction nomogram for prediction in children with wheeze.

As the relative contribution of each individual factor to the development of childhood asthma or wheeze might be small, it is of importance to consider the joint contribution of selected factors of significance. To achieve this goal, we constructed risk prediction nomogram models for childhood asthma and wheeze, respectively, by incorporating factors of significance selected by the logistic LASSO regression analyses (Figure 3). Furthermore, ROC analyses were performed to evaluate the discrimination ability of the nomogram model for childhood asthma, with decent results (AUC = 0.746, 95% CI: 0.7109–0.7803). Similarly, the nomogram model for the prediction of childhood wheeze (Figure 4) had higher prediction efficacy (AUC = 0.715, 95% CI: 0.6913–0.7388). The predictive accuracy of the nomograms was determined using calibration plots (Supplementary Figure 2A for asthma and Supplementary Figure 2B for wheeze), and the C-index were 0.728 and 0.707, respectively.

To better understand the utility of the nomogram model, an example was given here. Taking the risk prediction nomogram model for childhood asthma as an example: assuming a child aged 6 years old (14 points), with a history of allergic rhinitis (75 points), without a history of hay fever and eczema, using antibiotics within 1 month of birth (28 points), with vitamin D supplement duration over 1 year (0 points), with a family history of asthma (100 points), and being overweight (15 points), the probability of asthma for this child was estimated to be 75%.

### Internal Validation

The two constructed nomogram models were internally validated using bootstrap resampling with 100 replications. For asthma, the C-statistic was 0.728 in the derivation cohort, with good internal validation (bootstrap C-statistic of 0.736) and excellent calibration of predicted and observed risk. For wheeze, the C-statistic was 0.707 in the derivation cohort, and the bootstrap C-statistic was 0.708.

## Discussion

In the present study, *via* a large-scale cross-sectional survey, data from 9,529 Chinese preschool-aged children were collected to identify and characterize potential factors associated with childhood asthma and wheeze in China. By means of the logistic LASSO regression, which minimizes multicollinearity between variables, we separately identified seven and eight factors in a significant association with childhood asthma and wheeze. Importantly, the incorporation of these factors into nomogram models can robustly predict the risk of childhood asthma and wheeze, with decent accuracies. To the best of our knowledge, this is to date the first comprehensive research in China that has combined genetic, prenatal, perinatal, and postnatal factors to examine the possible association with asthma and wheeze among preschool-aged children.

Asthma is a consequence of complex gene environment interactions. It is widely recognized that family history plays a key role. As reported by a meta-analysis of 33 studies, maternal asthma increased offspring asthma risk to approximately three-fold greater than those free of maternal asthma (18). Consistently, in the present study, family history of asthma played a crucial part in predicting childhood asthma and wheeze. Meanwhile, the impact of the external environment on childhood asthma and wheeze is equally important. External factors, including maternal weight gain and obesity during pregnancy (19), childhood overweight and obesity (20), preterm delivery (21), breastfeeding duration (22), environmental tobacco smoke (23), and use of antibiotics (24), have been well-acknowledged to be associated with the risk of childhood asthma in agreement with the findings of the present survey. The implication of obesity in the development of childhood asthma is biologically plausible. There is evidence that obesity-related reduction in lung volume, changes in the hormone, dyslipidemia, and inflammatory mediators were identified as possible mechanisms behind the relationship between overweight and childhood asthma (25, 26). Meanwhile, we found premature exposure to antibiotics contributed to the susceptibility of childhood asthma. Early-life host–microbiome interactions resulted in the proper development of the immune system. Antibiotics affected microbial composition markedly, even transient perturbations during critical developmental periods may compromise both immune tolerance and inflammatory responses (27).

Several important findings specializing in the present study merit adequate discussion. First, we found the children with a history of allergic rhinitis, hay fever, and eczema were significantly associated with childhood asthma and wheeze. Recently, many studies have explored that the coexistence of eczema, rhinitis, and asthma in the same child was more common than expected, as 44% of the observed comorbidity at age 4 years and 50% at age 8 years (28), and several studies have shown that allergic rhinitis was a risk factor for subsequent wheezing onset (29), and uncontrolled asthma (30). In the CAPS cohort, children with atopic eczema were more likely than were those without atopic eczema to have a history of food allergies, allergic rhinitis, and current wheeze (31). Sensitization to allergens (atopy) is a key component of atopic diseases such as asthma and allergic rhinitis. However, some studies found that both IgE-mediated and non-IgE-mediated mechanisms were probably involved in their intimate connection (28).

Second, we found intake frequency of night meals among preschool-aged children was another noteworthy factor contributing to the development of childhood wheeze. In this survey, we found nearly 8.9% of children had night meals every day, and ~9.9% of children had night meals >3 times a week. It is well-known that eating too much food before bedtime can easily lead to esophageal reflux, especially for children with a more flaccid cardia and a less powerful esophageal sphincter. Gastrooesophageal reflux disease has been found in 25–80% of adults and children with asthma. Mechanisms include increased acid reflux during exacerbations with hyperinflation, microaspiration triggering neurogenic inflammation, and β2 agonists reducing lower esophageal sphincter pressure (32). More importantly, frequent late-night snacks make children overweight and obese and further increase the risk of developing wheeze and asthma. The same is suitable for long electronic screen time. Longer screen time is associated with a variety of health harms for children, with evidence strongest for adiposity, unhealthy diet (33). Altogether, reducing the frequency of late-night snacks and electronic screen time may be important lifestyle changes of interest to effectively reduce wheezing and asthma in children.

## Limitations

Several limitations should be acknowledged for the present study. First, this survey is cross-sectional in nature, which precludes further comments on the cause–effect relationship between selected factors of significance and childhood asthma or wheeze. Second, recall bias cannot be entirely excluded, as the majority of data were obtained *via* questionnaires. Third, diagnosis biases could not be avoided because the wheezing symptoms caused by a cold or other respiratory illness cannot be excluded.

## Conclusions

Taken together, our findings indicated that the development of asthma and wheeze among preschool-aged children was probably determined by the joint contribution of multiple factors including inherited, nutritional, unhealthy lifestyles, and history of allergic disease. More attention to poor feeding practices and lifestyles of preschool-aged children and active prevention of allergic diseases is needed for the prevention of childhood asthma. Further validation of our findings in other independent groups is necessary.

## Data Availability Statement

The original contributions presented in the study are included in the article/Supplementary Material, further inquiries can be directed to the corresponding author/s.

## Ethics Statement

The studies involving human participants were reviewed and approved by Institutional Review Boards of the China-Japan Friendship Hospital. Written informed consent to participate in this study was provided by the participants' legal guardian/next of kin.

## Author Contributions

KW, ZZ, and WN contributed to the conceptualization of the study. XD, MY, SW, BP, and QW involved in the data collection. MY, XD, and SW participated in the investigation. XD helped in the data detection. WN and XD performed the statistical analysis and involved in writing. All authors read and approved the final manuscript prior to submission.

## Conflict of Interest

The authors declare that the research was conducted in the absence of any commercial or financial relationships that could be construed as a potential conflict of interest.

## Publisher's Note

All claims expressed in this article are solely those of the authors and do not necessarily represent those of their affiliated organizations, or those of the publisher, the editors and the reviewers. Any product that may be evaluated in this article, or claim that may be made by its manufacturer, is not guaranteed or endorsed by the publisher.
