# Supplementary material for: Factors Associated With Childhood Asthma and Wheeze in Chinese Preschool-Aged Children
Source: Front Med (Lausanne). 2021 Nov 4;8:742581. doi: 10.3389/fmed.2021.742581 (PMC8599959; doi:10.3389/fmed.2021.742581)
Supplement: Supplementary file 1 [file Data_Sheet_1.docx]

**Table/Figure legends:**

**Table 1.** Baseline characteristics of study participants in this study.

**Table 2.** Baseline characteristics of study participants after Propensity Score Matching in this study.

**Table 3.** The estimated coefficients for logistic least absolute shrinkage and selection operator (LASSO) regression between candidate risk factors with asthma/wheeze.

**Figure 1.** Cross validation plot for the penalty term.

**Figure 2.** Prediction nomogram for prediction in children with asthma/wheeze.

**Supplementary Figure 1.** Plots for LASSO regression coefficients over different values of the penalty parameter.

**Supplymentary Figure 2.** Calculation curves of the nomogram for childhood asthma/wheeze.

**Supplementary Figure 1.** Plots for LASSO regression coefficients over different values of the penalty parameter. In (A), data shown are the enrolled factors that remained in the model of asthma; in (B), data shown are the enrolled factors that remained in the model of wheeze.

(A)


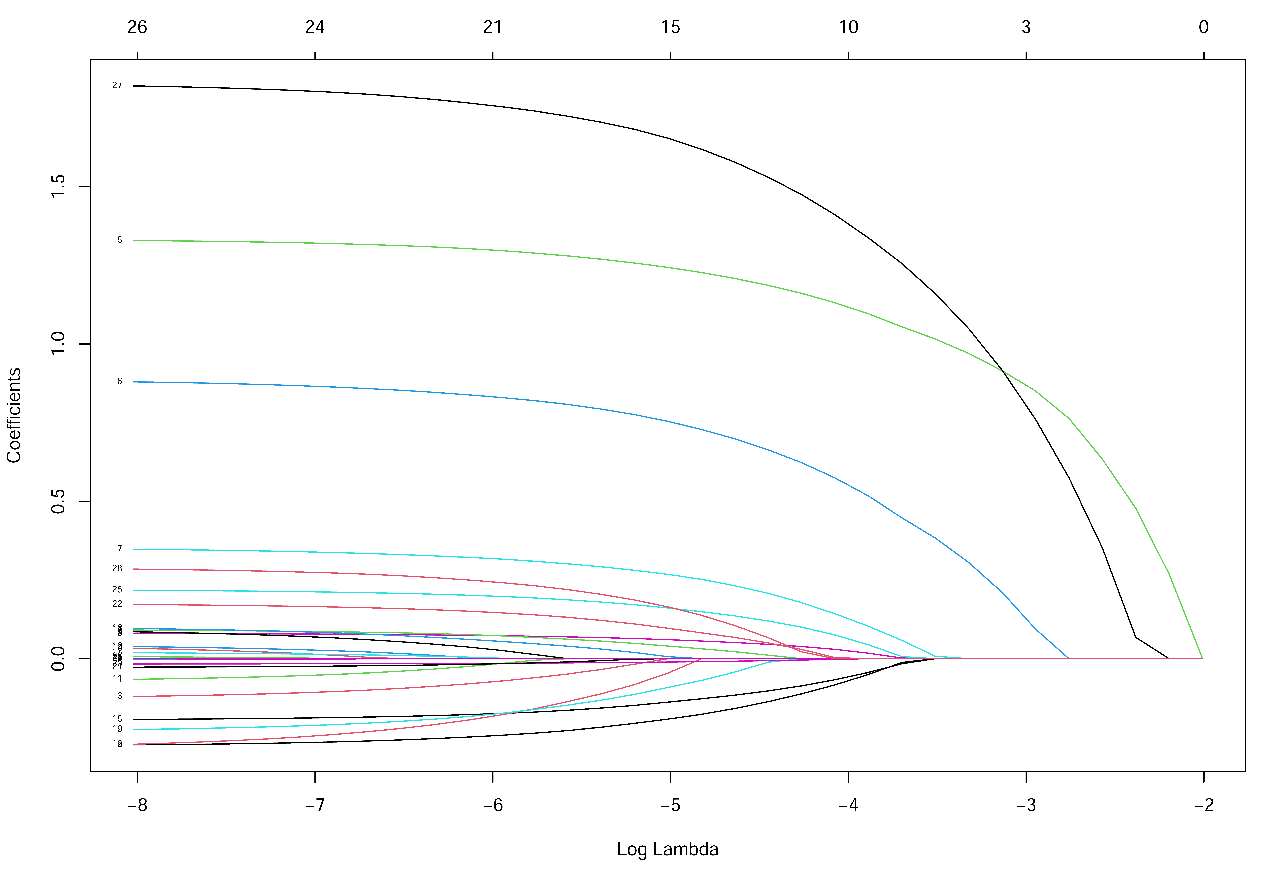


(B)


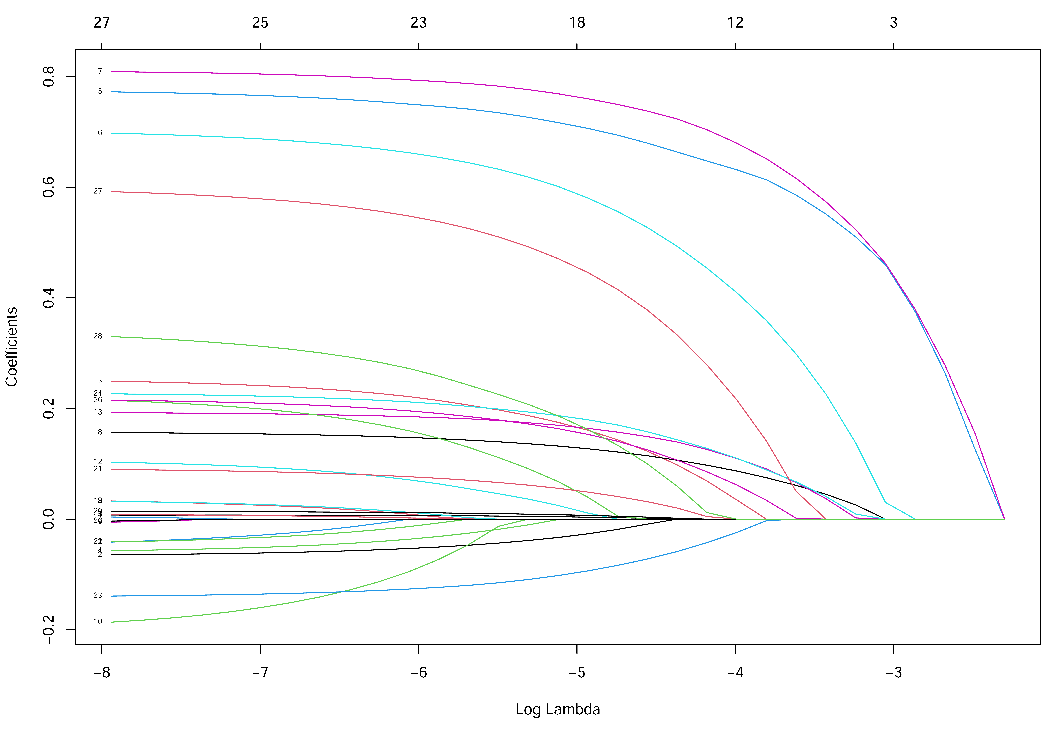


**Supplymentary Figure 2. (A)** Calculation curves of the nomogram for childhood asthma.





**Supplymentary Figure 2. (B)** Calculation curves of the nomogram for childhood wheeze.
